# 391. Patients Hospitalized for Acute Coronary Syndrome with Concomitant COVID-19 have Worse Clinical Outcomes

**DOI:** 10.1093/ofid/ofad500.461

**Published:** 2023-11-27

**Authors:** Deepali Boothankad Sharath, Leonid Khokhlov, Usha Thapa, Amr Aboelnasr, Leanne Pereira, Jose Improvola, Anar Patel

**Affiliations:** TriHealth Good Samaritan Hospital, Cincinnati, Ohio; TriHealth Good Samaritan Hospital, Cincinnati, Ohio; TriHealth Good Samaritan Hospital, Cincinnati, Ohio; TriHealth Good Samaritan Hospital, Cincinnati, Ohio; TriHealth Good Samaritan Hospital, Cincinnati, Ohio; Trihealth Heart and Vascular Institute, Cincinnati, Ohio; TriHealth Infectious Diseases, Cincinnati, Ohio

## Abstract

**Background:**

Since the onset of the global pandemic caused by COVID-19, there has been a multitude of studies that demonstrate the association between COVID-19 infection and adverse clinical outcomes. To date, there is a paucity of evidence showing how COVID-19 affects morbidity and mortality among patients admitted with cardiac events in large heterogeneous populations. We aimed to investigate the clinical outcomes in patients hospitalized with acute coronary syndrome (ACS) with co-existing COVID-19 infection using the National Inpatient Sample.

**Methods:**

We queried the Nationwide Inpatient Sample (NIS) database for the year 2020 for adult patients who were hospitalized with ACS with a concomitant diagnosis of COVID-19. The primary outcome was inpatient mortality. The secondary outcomes measured were cardiogenic shock, cardiac arrest, severe sepsis, invasive mechanical ventilation, length of stay (LOS), and total hospital cost. Multivariable logistic and Poisson regression analyses were used to estimate clinical outcomes. The p-value < 0.05 was significant.

**Results:**

There were 582,424 hospitalizations with ACS, of which 6,074 (1.1%) had COVID-19 infection. Patient characteristics are shown in Table 1. Patients with comorbid COVID-19 infection had significantly higher mortality and worse clinical outcomes (Table 2 and Figure 1).

**Figure:**
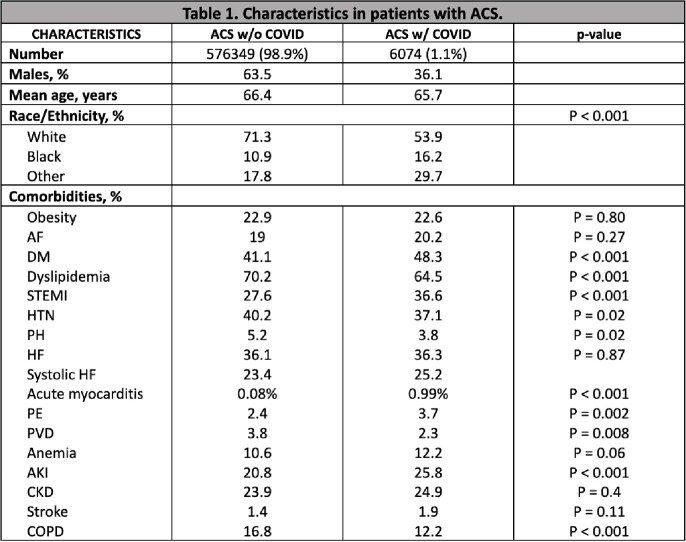

**Figure:**
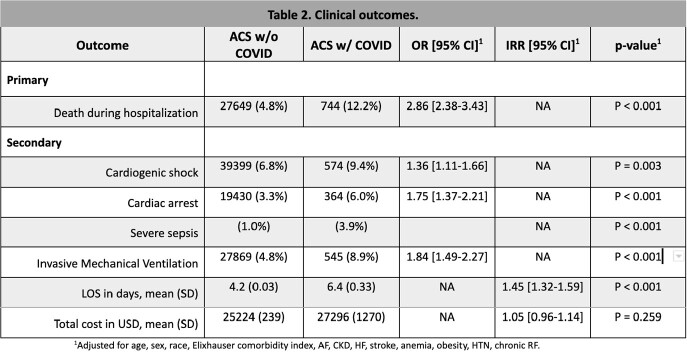

**Figure:**
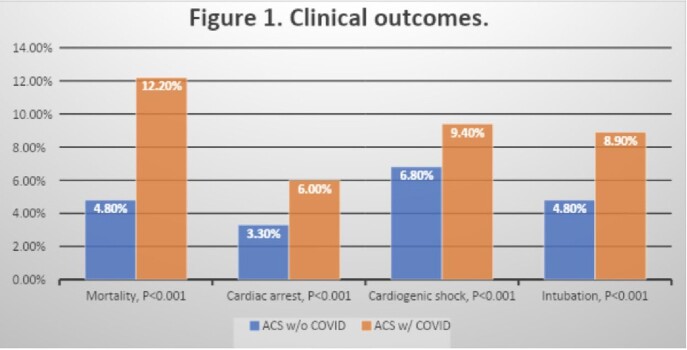

**Conclusion:**

Patients hospitalized with ACS and concomitant COVID-19 infection had a significantly increased risk for adverse clinical outcomes and higher resource utilization. They were younger, female, with more prevalent STEMI, acute myocarditis, PE, DM, AKI, and stroke. COVID-19 infection in the setting of ACS was associated with higher mortality and poor outcomes including cardiogenic shock and cardiac arrest as well as respiratory failure requiring mechanical ventilation. Further research to elucidate the correlation between COVID-19 infection and cardiac events is needed.

**Disclosures:**

**All Authors**: No reported disclosures

